# Supplementary material for: Pressure Induced Stability Enhancement of Cubic Nanostructured CeO2
Source: Nanomaterials (Basel). 2020 Mar 31;10(4):650. doi: 10.3390/nano10040650 (PMC7221606; doi:10.3390/nano10040650)
Supplement: Supplementary file 1 [file nanomaterials-10-00650-s001.pdf]

Supplementary

Pressure Induced Stability Enhancement of Cubic Nanostructured CeO_2

Mariano Andres Paulin [1,*], Gaston Garbarino [2,*], Ana Gabriela Leyva [,3,] Mohamed Mezouar [2] and Joaquin Sacanell [3,5]

[1] Laboratorio Argentino de Haces de Neutrones, Centro Atómico Bariloche, CNEA, Av. E. Bustillo 9500, San Carlos de Bariloche, Río Negro R8402AGP, Argentina

[2] European Synchrotron Radiation Facility, 71 Av. des Martyrs, Grenoble 38000, France; mezouar@esrf.fr

[3] Departamento de Física de la Materia Condensada, Centro Atómico Constituyentes, CNEA, Av. Gral. Paz 1499, San Martín, Buenos Aires 1650, Argentina leyva@cnea.gov.ar (A.G.L.); sacanell@tandar.cnea.gov.ar (J.S.) Escuela de Ciencia y Tecnología, Universidad Nacional de General San Martín, Alem 3901, San Martín (1650), Buenos Aires 1650, Argentina

[4] Instituto de Nanociencia y Nanotecnología, CNEA-CONICET, Av. Gral. Paz 1499, San Martín (1650), Buenos Aires 1650, Argentina

* Correspondence: mariano.paulin@cab.cnea.gov.ar (M.P.); gaston.garbarino@esrf.fr (G.G.); Tel.: +33-4 38 88-1987 (G.G.)

Received: 27 February 2020; Accepted: 24 March 2020; Published: date

'*In memory of Dr. Claudio Ferrero*'

1. Samples preparation and description

Ceria nanoparticles were prepared by the LIQUID-MIX [1] method using 99.99% Cerium nitrate from Alpha Aesar as precursor. Cerium nitrate was dissolved in milli-Q water and citric acid was added to the solution. This solution was then mixed in a heated plate until a foam was obtained and dried in a stove at 105 °C for two days. The dried foam was subsequently grinded with an agate mortar and finally calcined in air at T = 300 °C in a platinum melting pot in order to obtain a nano-crystalline CeO_2 powder (NANO). Samples with an average crystallite size of 4 nm were obtained.

A Ceria powder from NIST with particle size distribution of (380.6 ± 4.5) nm (MICRO) was also measured [2]. Finally, a ceria single crystal of 20 μm lateral dimension and ~10 μm thickness (CRYSTAL) has also been measured in order to extend in four orders of magnitude the analysis of particle size.

2. Ce^{3+} estimation on the NANO sample

At room pressure, the lattice parameter (a_{CUBIC}) can be expressed in terms of the ionic size as

$$\frac{\sqrt{3}}{4} a_{CUBIC} = r_{Ce^{4+}} + r_{O^{2-}} \tag{1}$$

where $r_{Ce^{4+}}$ and $r_{O^{2-}}$ are the ionic radii of Ce^{4+} and O^{2-} ions. The change in the lattice parameter due to the presence of Ce^{3+} can be then expressed as

$$\frac{\sqrt{3}}{4} (a_{NANO} - a_{MICRO}) = c\, [r_{Ce^{3+}} - r_{Ce^{4+}} + \frac{1}{4} (r_{Vo} - r_{O^{2-}})] \tag{2}$$

Where c is the Ce^{3+}/Ce^{4+} ratio, V_O represents an oxygen vacancy and then the V_O/O^{2-} ratio is $c/4$. The lattice parameters are a_{NANO} and a_{MICRO}, for the NANO and MICRO ceria respectively. We also used $r_{Ce^{3+}}$ = 0.1283 nm, $r_{Ce^{4+}}$ = 0.1098 nm, r_{V^0} = 0.138 nm and $r_{O^{2-}}$ = 0.124 nm [3]. Taking the a_{CUBIC} obtained at room pressure and the difference between a_{NANO} and a_{MICRO}.

3. Consideration of non-hydrostatic conditions

In order to clarify the origin of the discrepancy we considered the possible effect of non-hydrostatic conditions as proposed by A. K. Singh [4]. Following Singh's formalism in a cubic system, the strain produced by the deviatoric stress component due to non-hydrostatic compression will affect the measured lattice parameter $a_m(hkl)$ obtained for the Bragg peak (hkl).

$$a_m(hkl) = M_0 + M_1[3\Gamma(hkl)(1 - 3sin^2\theta)] \tag{3}$$

Where with some approximations [5]

$M_0 \approx a_p$, $M_1 = -a_p(\alpha St/3)$, $\Gamma(h\,k\,l) = {h^2k^2 + k^2l^2 + l^2h^2}/{h^2 + k^2 + l^2}$, $S = (S_{11} - S_{12} - S_{44}/2)$, t is the uniaxial stress component, S_{ij} are the elastic compliances and θ the diffraction angle. Thus the plot of $a_m(hkl)$ vs $3\Gamma(hkl)(1 - 3sin^2\theta)$, called gamma plot, would correspond to straight lines. These gamma plots were constructed for all the measured points for the NANO sample. Some representative ones are presented in Figure S1. As it is shown in this figure, the experimental points cannot be fitted with a straight line. The R^2 values varies between 0.2 and 0.6, indicating that the non-hydrostatic conditions are not the origin of the variation of the measured lattice parameters $a_m(hkl)$.

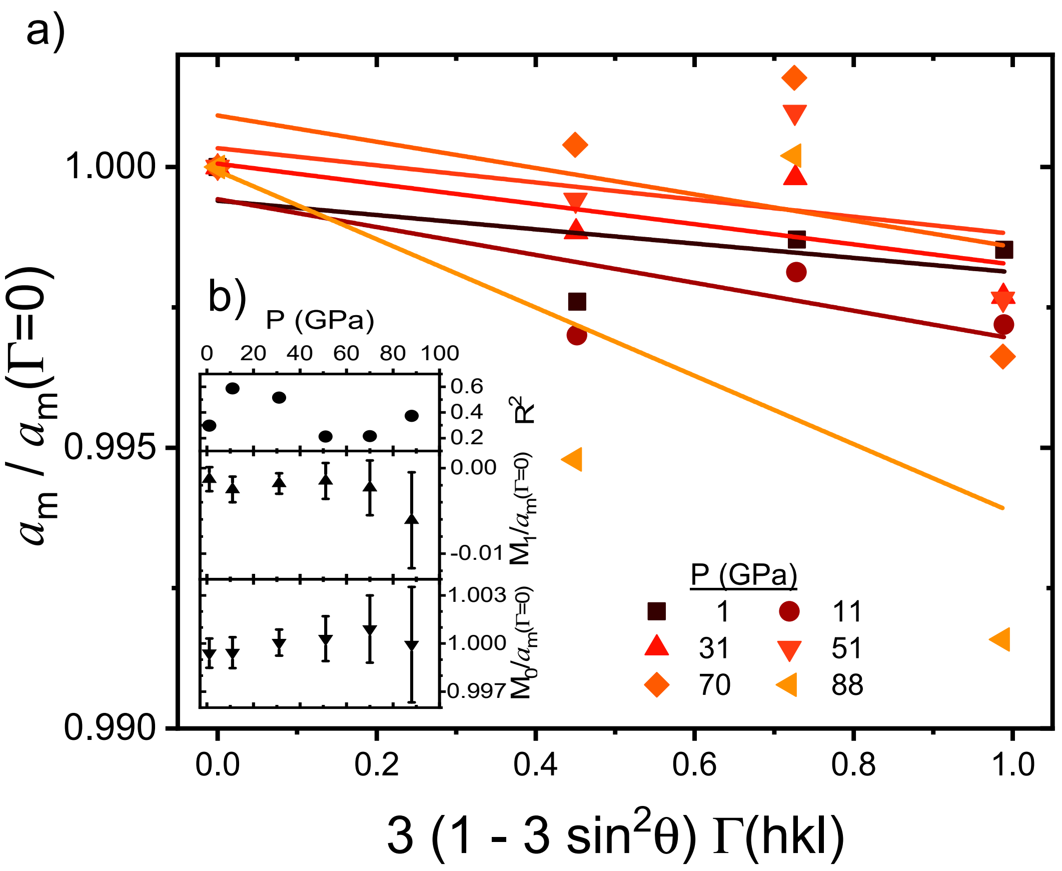

Figure S1: (**a**) Gamma plot for selected pressures for the NANO sample where the $a_m(hkl)$ is normalized using its value at abscise equal to 0, i.e. Γ = 0. (**b**) Pressure evolution of the obtained intercept (M_0/a_m (Γ = 0)), slope (M_1/a_m (Γ = 0)) and the coefficient of determination R^2.

Reference

1. Pechini M.P. Method of preparing lead and alkaline earth titanates and niobates and coating method using the same to form a capacitor. U.S. Patent 3,330,697, 11 July 1967.
2. *Quantitative Powder Diffraction Standard (SRM 674b) Consisting of Fine-Grained, High-Purity, Equi-Axial Grains That Are Not in An Aggregated State. Standard Reference Material from NIST, 674b, X-Ray Powder Diffraction Intensity Set*; NIST: Gaithersburg, US, 2011.
3. Shannon R.D.Revised Effective Ionic Radii and Systematic Studies of Interatomie Distances in Halides and Chaleogenides *Acta Cryst.* **1976**, *A32*, 751–767.
4. Singh A.K. The lattice strains in a specimen (cubic system) compressed nonhydrostatically in an opposed anvil device. *J. Appl. Phys.* **1993**, *73*, 4278–4286.
5. Singh A.K.; Kenichi T. Measurement and analysis of nonhydrostatic lattice strain component in niobium to 145 GPa under various fluid pressure-transmitting media. *J. Appl. Phys.* **2001**, *90*, 3269.
